# Supplementary figures and images for: A novel and evolutionarily conserved inhibitory circuit selectively regulates dentate gyrus mossy cell function
Source: Res Sq. 2025 Aug 22:rs.3.rs-7268106. Preprint. [Version 1] doi: 10.21203/rs.3.rs-7268106/v1 (PMC12393461; doi:10.21203/rs.3.rs-7268106/v1)

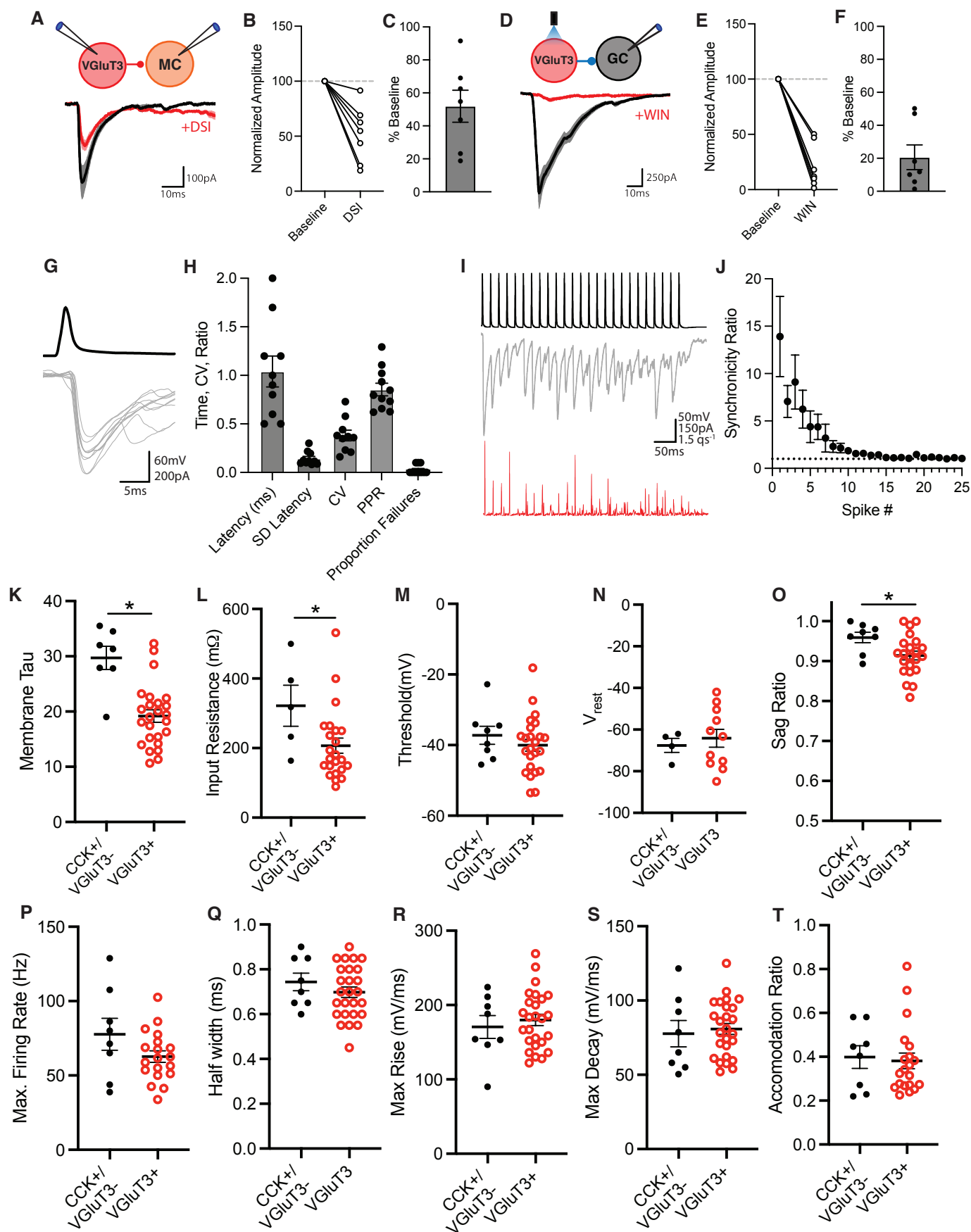

Supplemental Figure 1

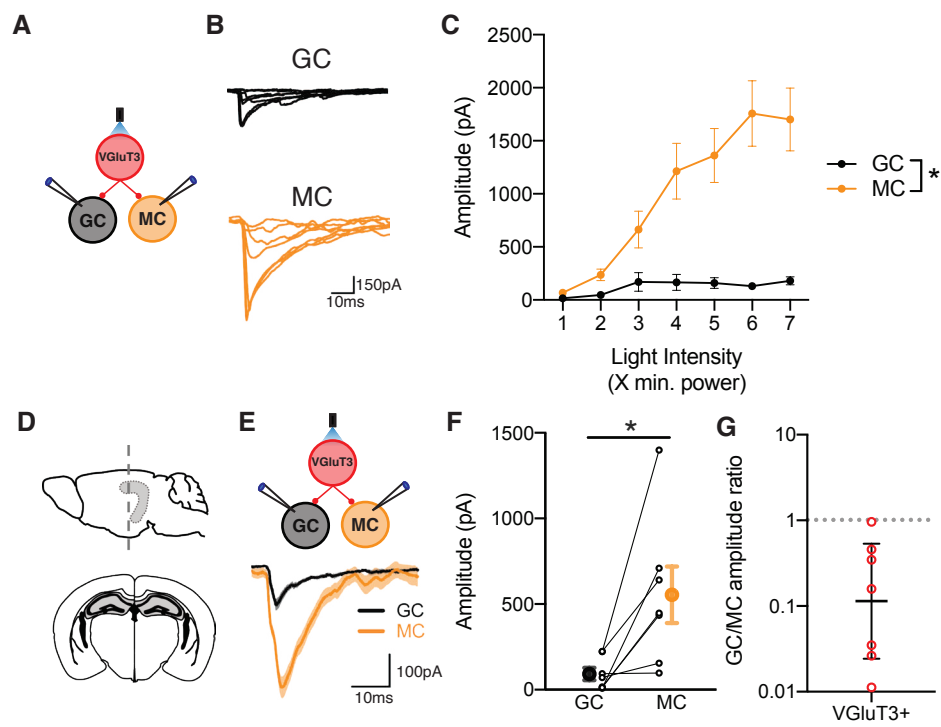

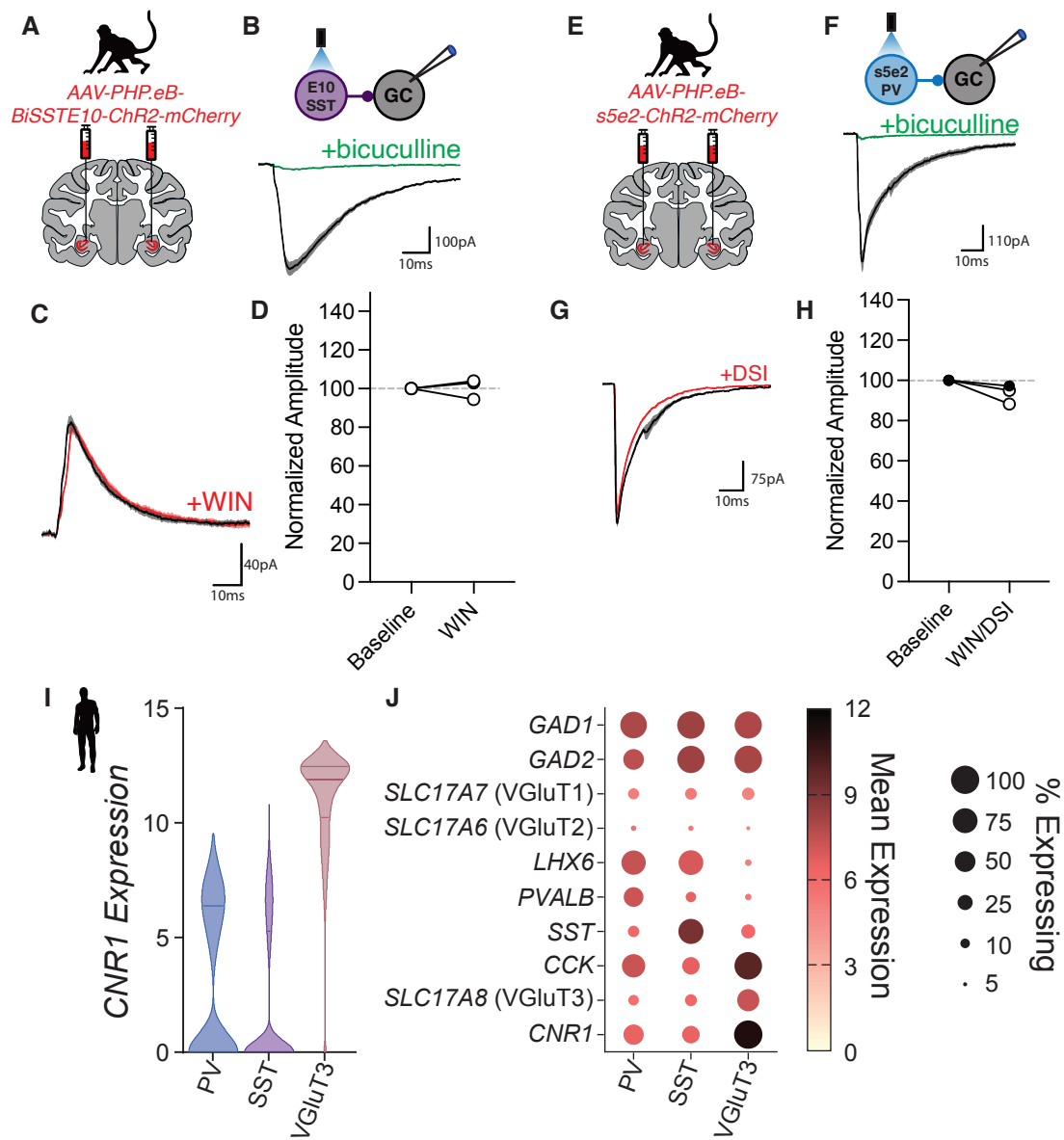

Supplemental Figure 3

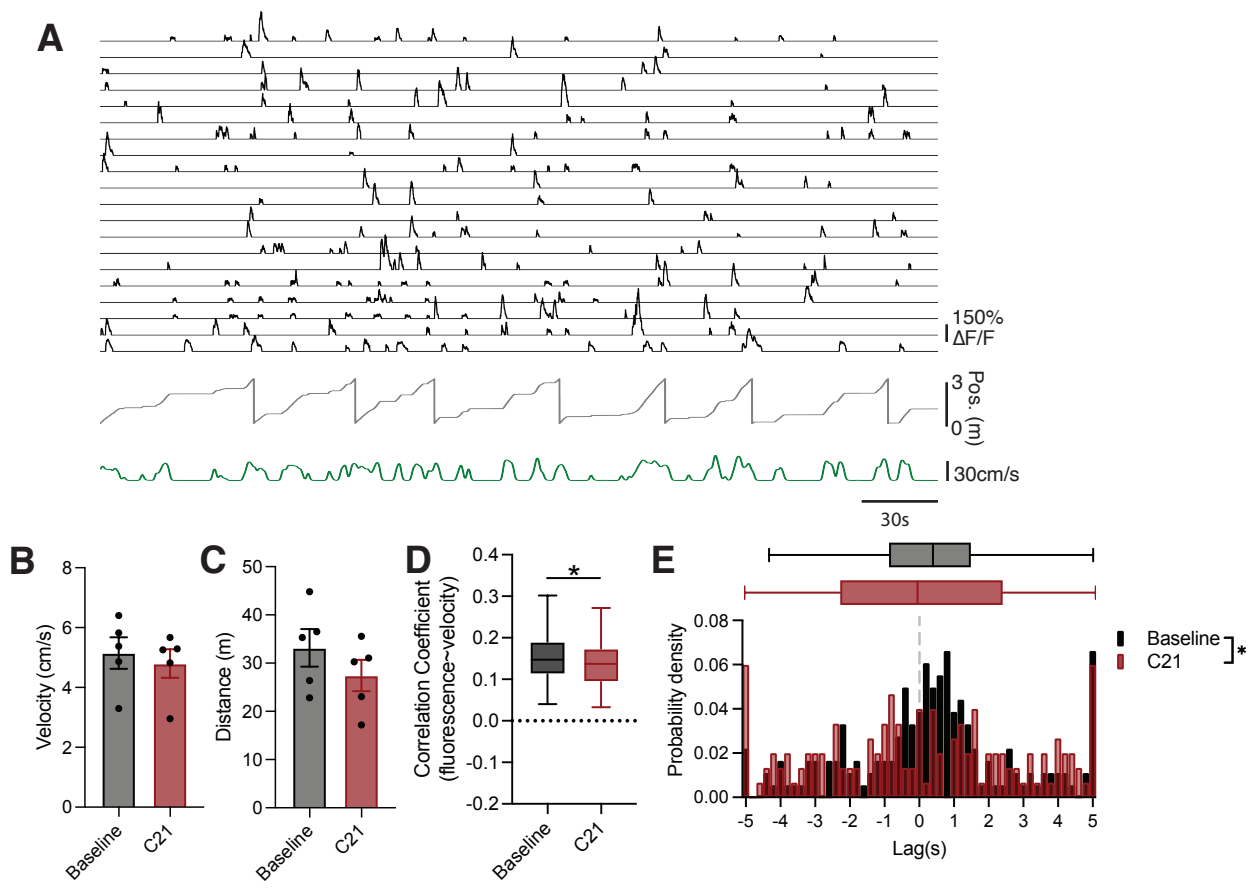

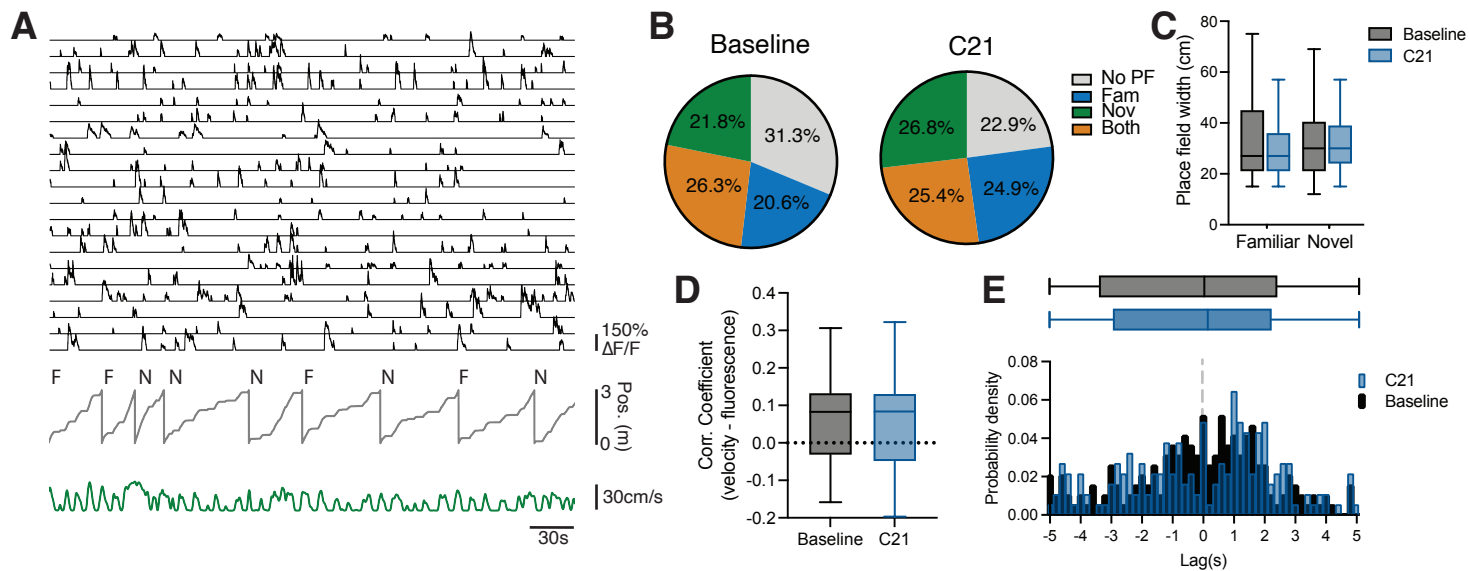

Supplemental Figure 5

Supplement: 1 — Supplemental Figure 1. Synaptic properties of DG VGluT3+ interneurons. A) Schematic of recording setup for paired VGluT3+ interneuron-MC recordings (top) and representative traces of uIPSC at baseline and after DSI (bottom). B) Graph showing normalized amplitude (as % of baseline) during baseline and after DSI. C) Group data showing average amplitude as a percentage of baseline after DSI (n=7 pairs, 3 mice; mean± s.e.m.). D) Schematic of recording setup for examining VGluT3+ interneuron leIPSCs simultaneously in MCs and GCs (top) and representative traces of leIPSCs in MCs during baseline and after bath application of WIN55,212–2 (bottom). E) Plot showing normalized amplitude (as % of baseline) during baseline and after WIN application. F) Group data displaying average leIPSC amplitude in MCs after WIN expressed as percentage of baseline (n=7 cells, 2 mice; mean± s.e.m.). G) Representative traces of VGluT3+ mediated uIPSC recorded in an MC. H) Group data on VGluT3+ interneuron synaptic latency, jitter (SD of latency), coefficient of variation (CV), paired-pulse ratio (PPR) and proportion of failures recorded in postsynaptic MC (n=10 pairs, 5 mice; mean± s.e.m.). I) Representative traces of presynaptic AP train in VGluT3+ interneuron (top), postsynaptic uIPSCs in MC during the train (middle) and release rate histogram (bottom). J) Plot show synchronicitiy ratio of VGluT3+ interneuron mediated uIPSCs across a 25-pulse train at 50 Hz. Dotted line indicates SR of 1 (n=10 cells, 5 mice). K-T) Plots showing passive and active electrophysiological properties for CCK+/VGluT3− and VGluT3+ interneurons in DG (*<0.05, t-test, mean± s.e.m.). Supplemental Figure 2. Optogenetic input-output relationship for VGluT3+ interneurons and VGluT3+ target selectivity in dorsal DG. A) Diagram of recording setup for recording VGluT3+ interneuron leIPSCs simultaneously in MCs and GCs. B) Representative traces of leIPSCs recording GCs (top) and MCs (bottom) across different light intensities. [file NIHPPRS7268106V1-supplement-1.pdf]
